# Neurocognitive Effects of Repetitive Transcranial Magnetic Stimulation in Adolescents with Major Depressive Disorder

**DOI:** 10.3389/fpsyt.2013.00165

**Published:** 2013-12-12

**Authors:** Christopher A. Wall, Paul E. Croarkin, Shawn M. McClintock, Lauren L. Murphy, Lorelei A. Bandel, Leslie A. Sim, Shirlene M. Sampson

**Affiliations:** ^1^Department of Psychiatry and Psychology, Mayo Clinic, Rochester, MN, USA; ^2^Division of Child and Adolescent Psychiatry, Mayo Clinic, Rochester, MN, USA; ^3^Neurocognitive Research Laboratory, Division of Brain Stimulation and Neurophysiology, Department of Psychiatry and Behavioral Sciences, Duke University School of Medicine, Durham, NC, USA; ^4^Department of Psychiatry, University of Texas Southwestern Medical Center, Dallas, TX, USA

**Keywords:** adolescents, depression, neurocognition, memory, learning, TMS

## Abstract

**Objectives:** It is estimated that 30–40% of adolescents with major depressive disorder (MDD) do not receive full benefit from current antidepressant therapies. Repetitive transcranial magnetic stimulation (rTMS) is a novel therapy approved by the US Food and Drug Administration to treat adults with MDD. Research suggests rTMS is not associated with adverse neurocognitive effects in adult populations; however, there is no documentation of its neurocognitive effects in adolescents. This is a secondary *post hoc* analysis of neurocognitive outcome in adolescents who were treated with open-label rTMS in two separate studies.

**Methods:** Eighteen patients (mean age, 16.2 ± 1.1 years; 11 females, 7 males) with MDD who failed to adequately respond to at least one antidepressant agent were enrolled in the study. Fourteen patients completed all 30 rTMS treatments (5 days/week, 120% of motor threshold, 10 Hz, 3,000 stimulations per session) applied to the left dorsolateral prefrontal cortex. Depression was rated using the Children’s Depression Rating Scale-Revised. Neurocognitive evaluation was performed at baseline and after completion of 30 rTMS treatments with the Children’s Auditory Verbal Learning Test (CAVLT) and Delis–Kaplan Executive Function System Trail Making Test.

**Results:** Over the course of 30 rTMS treatments, adolescents showed a substantial decrease in depression severity. Commensurate with improvement in depressive symptoms was a statistically significant improvement in memory and delayed verbal recall. Other learning and memory indices and executive function remained intact. Neither participants nor their family members reported clinically meaningful changes in neurocognitive function.

**Conclusion:** These preliminary findings suggest rTMS does not adversely impact neurocognitive functioning in adolescents and may provide subtle enhancement of verbal memory as measured by the CAVLT. Further controlled investigations with larger sample sizes and rigorous trial designs are warranted to confirm and extend these findings.

## Introduction

Repetitive transcranial magnetic stimulation (rTMS) is a novel treatment approach for medication-resistant patients with major depressive disorder (MDD). Repetitive TMS has been approved by the US Food and Drug Administration for the treatment of adults with MDD who fail to achieve satisfactory improvement from one prior adequate antidepressant treatment trial. Although several sham-controlled studies have indicated that rTMS is efficacious in adults with MDD (1–4), there have been few studies in adolescents. We recently reported results of an open-label pilot study that found rTMS to be a potentially effective adjunctive therapy for adolescents with treatment-resistant MDD (5). Adolescents showed statistically significant improvement in the Children’s Depression Rating Scale-Revised (CDRS-R) from baseline through the rTMS treatment series (30 sessions) and at 6-month follow-up. A second, recently completed replication trial in 10 adolescents revealed similar findings for clinical improvement in treatment completers (submitted). In both studies, assessments of cognitive functioning were performed at baseline and treatment completion.

A number of studies have indicated that rTMS treatment for MDD is not associated with adverse effects on neuropsychological functions such as attention, learning, and memory (2, 3, 6–9), but these investigations have only included adults. Not only have studies not shown any deterioration in neuropsychological functioning from rTMS, but several investigations have shown an improvement in neurocognitive function in adult patients with MDD. For example, in a sham-controlled study by Avery et al. (1), adults with MDD showed considerably improved performance on measures of attention, learning and memory, and cognitive flexibility following 10 sessions of 10 Hz rTMS applied to the left dorsolateral prefrontal cortex (L-DLPFC). Furthermore, two sham-controlled studies reported improvement in verbal memory performance as a result of multiple sessions of 10 Hz rTMS treatment to the L-DLPFC in adults with MDD (10, 11). Also, following multiple sessions of 10 Hz rTMS to the L-DLPFC in depressed adults, Fitzgerald et al. (12) found that there was significant improvement in neuropsychological function, including autobiographical memory; and Martis et al. (13) reported improvement in working memory and executive function. Recently, Luber and Lisanby described a review of over 60 TMS studies that reported “significant improvements in speed and accuracy in a variety of tasks involving perceptual, motor, and executive processing” (14).

In the present study we evaluated the neurocognitive effects of rTMS when used as an adjunctive treatment for adolescents with treatment-resistant MDD. We hypothesized that adolescents would demonstrate no difference in measures of memory, executive functioning, or auditory and visual learning tasks following a robust course of left-sided, high-frequency rTMS.

## Materials and Methods

### Participants

Participants were diagnostically assessed by a board-certified child and adolescent psychiatrists (Paul E. Croarkin and Christopher A. Wall). This included a comprehensive clinical evaluation and standardized diagnostic interview that utilized the Kiddie Schedule for Affective Disorders and Schizophrenia for School-Age Children – Present and Lifetime Version (K-SADS-PL) (15). At the time of enrollment, all participants were receiving active antidepressant treatment for an MDD episode according to the *Diagnostic and Statistical Manual of Mental Disorders*, Fourth Edition, Text Revision (*DSM-IV-TR*) (16). Clinically significant depressive symptoms were defined by CDRS-R (17) total score of at least 40 (*t* score >63). Participants included those with treatment failure/non-response to at least one adequate antidepressant trial [i.e., treated with stable selective serotonin reuptake inhibitor (SSRI) dose regimen for at least 6 weeks as defined by a score of ≥3 on the Antidepressant Treatment History Form] (18). All participants continued treatment with a stable dose of their pre-study antidepressant during the rTMS course. Participants in psychotherapy were ineligible if they had changed therapists, type of psychotherapy, or providers in the 4 weeks prior to rTMS initiation. Participants were allowed to continue previous sleep aids such as melatonin, trazodone, or diphenhydramine during treatment. Stimulants, antipsychotics, mood stabilizers, and tricyclic antidepressants were not permitted during the active treatment phase.

Patients with comorbid secondary diagnoses of dysthymia, attention-deficit/hyperactivity disorder, or anxiety disorders were eligible for enrollment. However, patients with schizophrenia, schizoaffective disorder, bipolar spectrum disorders, substance abuse or dependence, somatoform disorders, dissociative disorders, post-traumatic stress disorder, obsessive-compulsive disorder, eating disorders, mental retardation, or pervasive developmental disorder/autism spectrum disorders were excluded from participation. Medical exclusions included preexisting seizure disorders or active neurologic conditions (e.g., brain tumor, dyskinesias, or paralysis). The screening process included a urine toxicology screen for drugs of abuse and a urine pregnancy test. All participants and treaters wore earplugs during the sessions to minimize the risk of auditory threshold changes.

### Study overview

Both trials were prospective, open, multicenter pilot trial of active rTMS in adolescents with MDD confirmed by the K-SADS-PL. Both studies received institutional review board approval and were performed under United States Food and Drug Administration Investigational Device Exemptions: trial #1 – G060269 and trial #2 – G110091. All patients provided written informed assent, and parents provided written informed consent per institutional review board-approved guidelines. Recruitment, outcomes, and potential adverse effects were monitored by a Data and Safety Monitoring Board comprised of clinicians with no direct involvement in the study.

### rTMS procedures

Identification of the treatment site and stimulus dosing were based on previously defined techniques and guidelines noted in adult rTMS trials (4, 19). In both trials, the motor cortex was identified, using the rTMS machinery via a single pulse administered every 3–5 s, at the location that produced a localized contraction of the contralateral abductor pollicis brevis muscle. Once this site was defined, the resting motor threshold (MT) was determined using a computer-assisted maximum likelihood threshold-hunting algorithm (MT Assist, Neuronetics Inc., Malvern, PA, USA). Repeat MT determinations occurred at least once every 10 treatments to assess for possible changes that could produce safety issues due to changes in cortical excitability.

In trial #1, the L-DLPFC treatment location was determined by moving the treatment coil 5 cm anterior to the MT location along a left superior oblique plane (20). In trial #2, the L-DLPFC treatment location was determined via an MRI-based neurolocalization technique. The identified treatment site was then marked and spatial coordinates were recorded with a mechanical coil positioning system to ensure reproducibility of the coil placement.

In both trials, a total of 30 treatments were administered across a range of 6–8 weeks. This range was chosen for potential variation in patient schedules related to school and family events. Thus, each patient was offered a total of 40 treatment opportunities in which to complete 30 treatments. Each treatment was titrated to 120% of calculated MT, at a frequency of 10 Hz, with stimulus train duration of 4 s and an inter-train interval of 26 s, for a total of 3,000 stimulations per treatment session. In trial #1, rTMS was delivered using the Neuronetics Model 2100 Therapy System; in trial #2 treatments were delivered using the NeuroStar System (Neuronetics, Inc., Malvern, PA, USA).

### Neurocognitive assessments

Neurocognitive testing was administered by trained psychometrists at baseline and upon completion of the active rTMS treatments. Testing was typically performed in the afternoon hours, although not universally due to scheduling accommodations related to subject school obligations, family work hours, and clinician/psychometrist availability. A doctorate-level child psychologist (Leslie A. Sim) analyzed the results. The neurocognitive battery was tailored to assess a variety of neurocognitive domains including psychomotor speed, simple attention, learning, memory, and executive function. Specifically, the battery included the Children’s Auditory Verbal Learning Test-2 (CAVLT-2) (21) and the Delis–Kaplan Executive Function System (D-KEFS) (22) Trail Making Test.

The CAVLT-2 (21) is a measure designed to quantify a child’s (ages 6.6–17.11) verbal learning and memory abilities. The measure is comprised of a 16-item word list that is administered across five trials, and the participant is asked to recall the words after each trial. A different set of words is then presented and the participant is asked to immediately recall the items from the new list (Interference Trial). Following the interference list, the participant is instructed to recall as many items as possible from the original list (immediate recall). Following a 15 min delay, the participant is asked to recall the original list for a final time (delayed recall). Finally, the participant is presented with a 32-item word list and asked to recognize the 16 words from the original list (Recognition Trial). The CAVLT-2 yields multiple indices of learning and memory, including immediate memory span, level of learning, immediate recall, delayed recall, recognition accuracy, and total intrusions.

The D-KEFS Trail Making Test is used to assess components of cognitive flexibility on a visual-motor sequencing task (23). It consists of five conditions: (a) Visual Scanning, (b) Number Sequencing, (c) Letter Sequencing, (d) Motor Speed, and (e) Number-Letter Switching. The D-KEFS Trail Making Test was selected to assess aspects of executive function, particularly cognitive flexibility, as it is influenced by mood and anxiety states. Importantly, the D-KEFS has been normed for the age group of children in this trial and provides standardized scores (24). Based on adequate reliability and validity of these tests along with appropriate developmental normative data, these neuropsychological measures (CAVLT-2 and D-KEFS TMT) were thought to be developmentally appropriate tools to assess subtle changes in cognitive function of adolescents receiving rTMS treatments.

Safety and participant comfort were assessed and recorded before and after each study visit with prompted opportunities to report adverse events.

### Statistical analysis

The primary aim of this study was to assess whether adjunctive rTMS is a safe and feasible treatment approach in adolescents. This question was evaluated by neurocognitive assessments that occurred at baseline and immediately following treatment number 30. Within patient changes from baseline to treatment completion were examined with comparative statistics.

Neurocognitive measurements obtained at baseline and immediately following treatment were summarized using mean and standard deviation (±SD). The paired *t*-test was used to assess whether scores changed significantly from baseline to end of treatment. For these analyses, two-tailed *P*-values of ≤0.05 were considered statistically significant. In addition to the primary analyses which included all enrolled subjects, a subset analysis was performed that was restricted to subjects who completed treatment. All analyses were performed using SAS software, version 9.2 (SAS Institute Inc., Cary, NC, USA).

## Results

**Figure 1:**
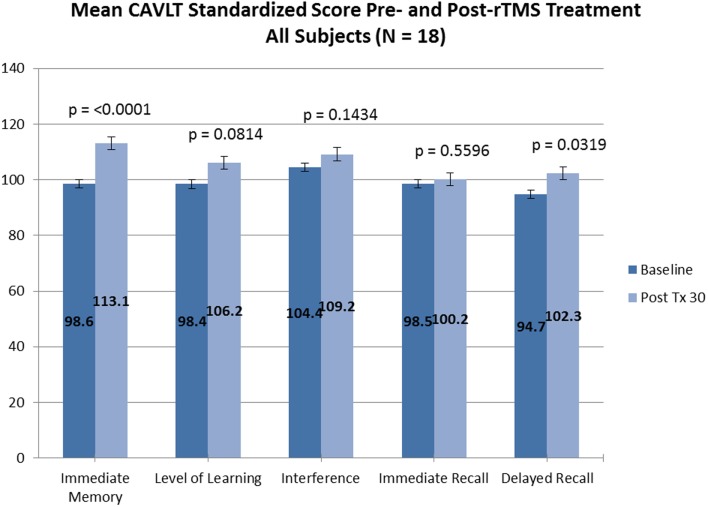
**CAVLT results (all participants)**.

**Figure 2:**
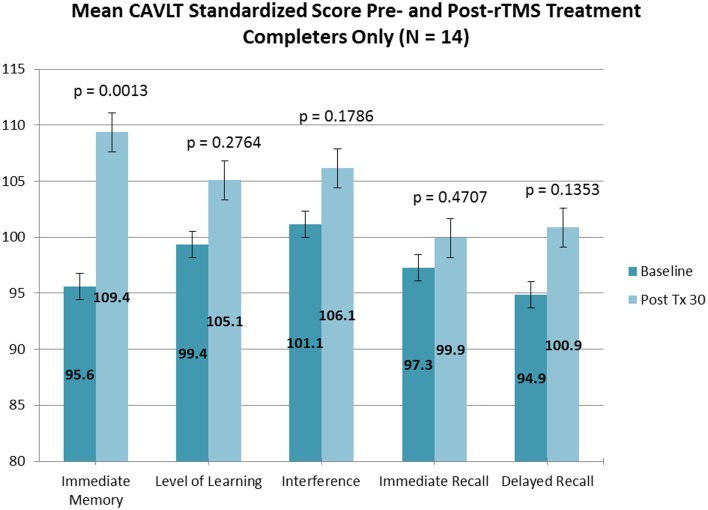
**CAVLT results (completers only)**.

Of the 18 adolescents enrolled in both trials, 14 adolescents (5 males and 9 females; ages 13.9–17.8 years; mean age, 16.3 ± 1.1 years) completed the entire rTMS treatment course (clinicaltrials.gov Identifier: NCT00587639). Fourteen out of 14 adolescents who completed the entire treatment course also completed neurocognitive testing at baseline and treatment completion. Subjectively, no reportable changes in memory, cognitive functioning, or attention were noted by any of the participating adolescents or their families. Objectively, subtle but statistically significant improvement was observed in immediate memory and delayed recall as measured by the CAVLT when measured in all study participants (Table 1; Figure 1) and participants who completed all 30 rTMS sessions of the treatment protocol (Table 2; Figure 2). All other CAVLT indices of learning and memory (interference, immediate recall, or level of learning) remained stable over the course of treatment (Table 1).

**Table 1:** **Children’s Auditory Verbal Learning Task (CAVLT) results (all participants)**.

CAVLT subscales		*N*	Score	Change from baseline	Change	*P*-value	Cohen’s *d*
			Mean	Mean (SD)	(95% CI)	
Immediate Memory Scale	BL	18	98.6	–	–	–	–
	PT	18	113.1	14.5 (12.0)	(8.5, 20.5)	<0.0001	0.81
Level of learning	BL	18	98.4	–	–	–	–
	PT	18	106.2	7.7 (17.7)	(−1.1, 16.5)	0.0814	0.37
Interference	BL	18	104.4	–	–	–	–
	PT	18	109.2	4.7 (13.1)	(−1.8, 11.2)	0.1434	0.30
Immediate recall	BL	18	98.5	–	–	–	–
	PT	18	100.2	1.7 (11.9)	(−4.2, 7.6)	0.5596	0.08
Delayed recall	BL	18	94.7	–	–		
	PT	18	102.3	7.6 (13.8)	(0.7, 14.5)	0.0319	0.33

*BL, baseline; PT, post-treatment*.

**Table 2:** **Children’s Auditory Verbal Learning Task (CAVLT) results (treatment completers)**.

CAVLT subscales		*N*	Score	Change from baseline	Change	*P*-value	Cohen’s *d*
			Mean	Mean (SD)	(95% CI)	
Immediate Memory Scale	BL	14	95.6	–	–	–	–
	PT	14	109.4	13.8 (12.6)	(6.5, 21.1)	0.0013	0.77
Level of learning	BL	14	99.4	–	–	–	–
	PT	14	105.1	5.7 (18.8)	(−5.2, 16.6)	0.2764	0.25
Interference	BL	14	101.1	–	–	–	–
	PT	14	106.1	5.0 (13.2)	(−2.6, 12.6)	0.1786	0.31
Immediate recall	BL	14	97.3	–	–	–	–
	PT	14	99.9	2.6 (13.3)	(−5.1, 10.3)	0.4707	0.11
Delayed recall	BL	14	94.9	–	–		
	PT	14	100.9	6.0 (14.1)	(−2.1, 14.1)	0.1353	0.24

*BL, baseline; PT, post-treatment*.

**Figure 3:**
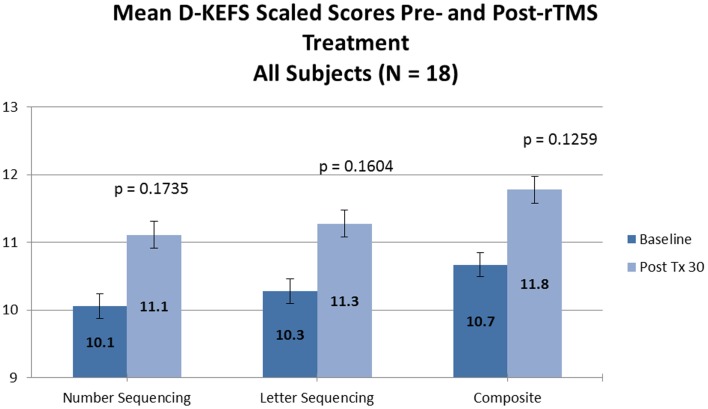
**D-KEFS results (all participants)**.

**Figure 4:**
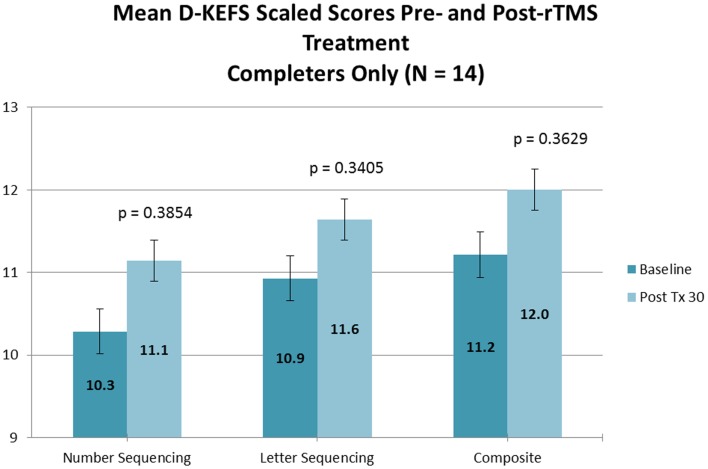
**D-KEFS results (completers only)**.

No significant changes were noted on the D-KEFS Trail Making Test indices from baseline to treatment completion for either the all participant group (Table 3; Figure 3) or the treatment completers only group (Table 4; Figure 4).

**Table 3:** **Delis–Kaplan Executive Function System (D-KEFS) Trail Making Test results (all participants)**.

D-KEFS subscales		*N*	Score	Change from baseline	Change	*P*-value	Cohen’s *d*
			Mean	Mean (SD)	(95% CI)	
Number sequencing	BL	18	10.1	–	–	–	–
	PT	18	11.1	1.1 (3.2)	(−0.5, 2.7)	0.1735	0.38
Letter sequencing	BL	18	10.3	–	–	–	–
	PT	18	11.3	1.0 (2.9)	(−0.4, 2.4)	0.1604	0.42
Composite score	BL	18	10.7	–	–	–	–
	PT	18	11.8	1.1 (2.9)	(−0.4, 2.6)	0.1259	0.43

**Table 4:** **Delis–Kaplan Executive Function System (D-KEFS) Trail Making Test results (treatment completers)**.

D-KEFS subscales		*N*	Score	Change from baseline	Change	*P*-value	Cohen’s *d*
			Mean	Mean (SD)	(95% CI)	
Number Sequencing	BL	14	10.3	–	–	–	–
	PT	14	11.1	0.9 (3.6)	(−1.2, 3.0)	0.3854	0.28
Letter sequencing	BL	14	10.9	–	–	–	–
	PT	14	11.6	0.7 (2.7)	(−0.9, 2.3)	0.3405	0.31
Composite score	BL	14	11.2	–	–	–	–
	PT	14	12.0	0.8 (3.1)	(−1.0, 2.6)	0.3629	0.30

## Discussion

To our knowledge, this is the first report on neurocognitive outcomes within a clinical trial of rTMS in depressed adolescents. The neurocognitive safety findings of this case series of high-frequency rTMS in treatment-resistant depressed adolescents are consistent with previously reported findings in clinical trials of rTMS in adults with psychiatric illness. Previous adult trials have frequently included cognitive assessments that demonstrated rTMS to have no adverse effects on cognitive functions (2, 3, 6–9, 25). Interestingly, a number of these clinical trials in adults have shown time-limited improvements in various aspects of cognitive function, mainly in attention, concentration, working memory, and processing speed (10–13). Similarly, modest improvements in attention, and verbal and learning and memory were observed in this cohort of adolescents.

## Limitations

Clearly, these findings must be interpreted with caution due to the small total number of participants and the lack of a control group. However, if there was a distinct pattern of clinically and psychometrically meaningful adverse cognitive effects – as could be found in a robust course of electroconvulsive therapy – we would expect to see these findings even in this small group of participants. It is reassuring to note that none of the participants or their family members described any impairments (or marked improvements) in learning, memory, or other untoward cognitive effects.

## Conclusion

Collectively, the findings of this study combined with our prior clinical findings suggest that rTMS may be a safe, feasible, and potentially efficacious adjunctive therapy for adolescents with MDD given the lack of negative changes in cognitive functioning and reduced overall side-effect burden (4, 5). Future studies of rTMS in adolescents will need to monitor for cognitive changes, which would benefit from the use of a comprehensive, standardized, and validated neurocognitive battery that will be sensitive to cognitive changes, particularly in those cognitive domains essential for continued academic maturation and instrumental activities of daily living. Such a neurocognitive battery should assess domains of intellectual ability, processing speed, attention, learning and memory, working memory, and executive function. Indeed, Semkovska and McLoughlin recommended the development of standardized and validated tools to measure the ability to recall autobiographical events in patients with depression (26). Such a measure would be of particular value in the adolescent population.

## Conflict of Interest Statement

Study 1 was funded by an American Academy of Child and Adolescent Psychiatry/Eli Lilly Pilot Research Award (Dr. Christopher A. Wall); a Small Grant Award from the Department of Psychiatry and Psychology, Mayo Clinic (Dr. Christopher A. Wall); and a Stanley Medical Research Institute Center Grant (Dr. Paul E. Croarkin). For Study 2 Dr. Christopher A. Wall received funding via funded by the Klingenstein Third Generation Foundation Fellowship in Child and Adolescent Depression; the Mayo Clinic CReFF award and Mayo Clinic Kids’ Cup scholarship. Funding was also provided by the National Institute of Mental Health (K23 MH087739, Dr. Shawn M. McClintock). Neuronetics, Malvern, PA, USA provided equipment support, but had no involvement in the protocol design or analysis. The other co-authors declare that the research was conducted in the absence of any commercial or financial relationships that could be construed as a potential conflict of interest.
